# Nephrology co-management versus primary care solo management for early chronic kidney disease: a retrospective cross-sectional analysis

**DOI:** 10.1186/s12882-015-0154-x

**Published:** 2015-10-12

**Authors:** Lipika Samal, Adam Wright, Sushrut S. Waikar, Jeffrey A. Linder

**Affiliations:** Division of General Medicine and Primary Care, Brigham and Women’s Hospital, 1620 Tremont St., Boston, MA 02120-1613 USA; Harvard Medical School, Boston, MA 02120 USA; Renal Division, Brigham and Women’s Hospital, 75 Francis St., Boston, MA 02120 USA

**Keywords:** Primary care, Nephrology, Quality of healthcare, Ambulatory care, Kidney diseases

## Abstract

**Background:**

Primary care physicians (PCPs) typically manage early chronic kidney disease (CKD), but recent guidelines recommend nephrology co-management for some patients with stage 3 CKD and all patients with stage 4 CKD. We sought to compare quality of care for co-managed patients to solo managed patients.

**Methods:**

We conducted a retrospective cross-sectional analysis. Patients included in the study were adults who visited a PCP during 2009 with laboratory evidence of CKD in the preceding two years, defined as two estimated glomerular filtration rates (eGFR) between 15–59 mL/min/1.73 m^2^ separated by 90 days. We assessed process measures (serum eGFR test, urine protein/albumin test, angiotensin converting enzyme inhibitor or angiotensin receptor blocker [ACE/ARB] prescription, and several tests monitoring for complications) and intermediate clinical outcomes (mean blood pressure and blood pressure control) and performed subgroup analyses by CKD stage.

**Results:**

Of 3118 patients, 11 % were co-managed by a nephrologist. Co-management was associated with younger age (69 vs. 74 years), male gender (46 % vs. 34 %), minority race/ethnicity (black 32 % vs. 22 %; Hispanic 13 % vs. 8 %), hypertension (75 % vs. 66 %), diabetes (42 % vs. 26 %), and more PCP visits (5.0 vs. 3.9; *p* < 0.001 for all comparisons). After adjustment, co-management was associated with serum eGFR test (98 % vs. 94 %, *p* = <0.0001), urine protein/albumin test (82 % vs 36 %, *p* < 0.0001), and ACE/ARB prescription (77 % vs. 69 %, *p* = 0.03). Co-management was associated with monitoring for anemia and metabolic bone disease, but was not associated with lipid monitoring, differences in mean blood pressure (133/69 mmHg vs. 131/70 mmHg, *p* > 0.50) or blood pressure control. A subgroup analysis of Stage 4 CKD patients did not show a significant association between co-management and ACE/ARB prescription (80 % vs. 73 %, *p* = 0.26).

**Conclusion:**

For stage 3 and 4 CKD patients, nephrology co-management was associated with increased stage-appropriate monitoring and ACE/ARB prescribing, but not improved blood pressure control.

## Background

Twenty-six million Americans have chronic kidney disease (CKD). Approximately 90 % of CKD patients are in an early stage of the disease and are typically cared for by primary care physicians (PCPs). U.S. and international guidelines recommend that physicians monitor for progression, monitor for complications, prescribe medications to delay progression, and focus on cardiovascular risk modification, all of which are within a PCP’s scope of practice [1]. Recent international guidelines, however,  recommend nephrology co-management for some patients with stage 3 CKD and all patients with stage 4 CKD, based on the assumption that co-management lessens complications, delays renal failure, and decreases mortality [2]. While nephrologist involvement is associated with decreased mortality for the small group of patients who progress to end stage renal disease and need renal replacement therapy [3, 4], we do not know whether co-management of a larger proportion of stage 3 patients and all stage 4 patients will improve quality of care. To assess the relationship between nephrology co-management and quality of care, we conducted a retrospective cross-sectional analysis of electronic health record (EHR) data for patients with stage 3 and 4 CKD, comparing co-managed patients to solo managed patients.

## Methods

### Study population and setting

After receiving approval from the Partners Healthcare Human Research Committee and being granted waiver of consent, we electronically screened all patients who had at least one visit to a PCP at one of 12 primary care clinics in the Brigham and Women’s Primary Care Practice Based Research Network during 2009. This network includes hospital-based and community-based clinics, two of which are federally qualified health centers. We included adult patients with stage 3 or 4 CKD, defined as two past estimated glomerular filtration rates (eGFR) between 15–60 mL/min/1.73 m^2^, separated by 90 days and collected during routine clinical care between January 1, 2007 to December 31, 2008 [5]. This approach allowed us to identify a cohort of patients who had CKD at the beginning of calendar year 2009. We excluded patients with documented end-stage renal disease.

### Data source

Data sources were billing data and EHR data. The practices have used the home-grown, CCHIT-certified, Longitudinal Medical Record EHR since July 2000. We used billing data, rather than EHR data, to identify clinic visits because the EHR notes do not specify whether nephrologist consultations were inpatient or outpatient. For the outcomes, we used EHR data. We queried the EHR for laboratory results, medications, and blood pressure measurements from 2009. PCPs prescribed all medications through the EHR and medications which were not prescribed by PCPs were included on the medication list as well. All outpatient vital signs were recorded in the EHR. Covariates such as socio-demographic data were collected during registration and updated periodically in the EHR. Our data was collected in 2009 because a large-scale EHR intervention was implemented in 2010 to increase recognition of CKD [6].

### Exposure, outcomes, and covariates

We defined the exposure, nephrology co-management, as at least one outpatient visit with a nephrologist over the course of the year 2009 according to billing data. The quality of care outcomes were derived from nationally accepted guidelines [5, 7]. We examined the following outcomes: 1) annual serum eGFR test (a measurement during the year 2009, in addition to the two measurements over 2007–2008 that were used for inclusion), 2) annual urine albumin/protein test, 3) an angiotensin converting enzyme inhibitor or angiotensin receptor blocker (ACE/ARB) prescription, 4) annual LDL test, 5) annual serum hemoglobin/hematocrit test, 6) annual serum calcium test, 7) annual serum phosphorus test, 8) annual serum parathyroid test for patients with stage 4 CKD, 9) mean blood pressure (BP; using last value for SBP and last value for DBP), and 10) BP control.

For the urine protein monitoring outcome, we included several tests: urine total protein, microalbumin, and albumin-to-creatinine ratio. We used medication list data to determine whether an ACE/ARB was listed as an active medication during 2009. We used the most recent recorded BP for BP outcomes. We examined hypertension by assessing mean BP and BP control according to two definitions: blood pressure ≤ 130/80 mmHg and ≤ 140/90 mmHg, due to differing opinions about the appropriate target for non-diabetic kidney disease in the absence of proteinuria [7–10].

We stratified CKD patients into stage 3 (two measurements of eGFR 30–59 mL/min/1.73 m^2^) and stage 4 (two measurements of eGFR 16–29 mL/min/1.73 m^2^). We examined tests to monitor for complications of CKD differently for stage 3 and stage 4 patients [11–14]. For stage 3 patients we examined 1) annual serum hemoglobin test, 2) annual serum calcium test and 3) annual serum phosphorus test. For stage 4 patients, we additionally examined annual serum parathyroid hormone test.

We examined several potential confounders of the relationship between co-management and outcomes by first assessing association between the potential confounder and co-management. We examined socio-demographic variables (age, gender and race), severity of disease as measured by eGFR, and co-morbid diabetes and hypertension, and the frequency of PCP visits. We hypothesized that PCP diagnosis of CKD could be related to, or on the causal pathway to, referral and so we examined the impact of adjusting for this variable in fully adjusted models.

### Statistical analysis

We examined the association of nephrology co-management and outcomes for stage 3 and stage 4 CKD patients and then performed subgroup analyses stratified by CKD stage.

We first analyzed the data assuming that the outcome was independent and then repeated the analyses accounting for clustering by PCP. Since we found similar results, we present all results accounting for clustering by PCP. We used multivariable logistic and linear regression to adjust for potential confounders and to produce weighted estimates (weighted to adjust for covariates and to account for clustering using the LSMEANS feature of the GENMOD procedure in SAS, SAS Institute, Cary, NC).

## Results and discussion

### Patient characteristics

Of the 79,605 patients who made a visit to a PCP in 2009, we identified 3,118 patients (4 %) with stage 3 or 4 CKD. Patients had a mean age of 74 years and were predominantly female (Table 1). Race/ethnicity rates were 67 % White, 23 % Black, 8 % Hispanic, and 1.5 % Asian. The average eGFR was 46 mL/min/1.73 m^2^, 27 % had diabetes, 67 % had hypertension, and, on average, patients saw their PCP 4 times during the year.

**Table 1:** Characteristics of stage 3 and stage 4 CKD patients and association with nephrology co-management

Patient characteristics	Total (*N* = 3118)	Nephrology co-management (*n* = 341)	Solo PCP management (*n* = 2777)	*p* value
Age, mean (SD)	74 (12)	69 (13)	74 (12)	*P* <0.0001
Male gender, N (%)	1108 (36 %)	155 (46 %)	953 (34 %)	*P* <0.0001
Race/Ethnicity, N (%)				*P* <0.0001
White	2060 (67 %)	177 (53 %)	1883 (69 %)	
Black	716 (23 %)	108 (32 %)	608 (22 %)	
Hispanic	253 (8 %)	45 (13 %)	208 (8 %)	
Asian	46 (2 %)	7 (2 %)	39 (1 %)	
Serum Creatinine, mean (SD)	1.46 (0.8)	2.28 (1.3)	1.36 (0.6)	*P* <0.0001
eGFR (average of 2 values), mean (SD)	46.0 (9.89)	35.6 (11.1)	47.3 (8.92)	*P* <0.0001
CKD Stage, N (%) (based on average of 2 values)				
3a (eGFR 45–59 mL/min/1.73 m^2^)	1929 (62 %)	80 (23 %)	1849 (67 %)	
3b (eGFR 30–44 mL/min/1.73 m^2^)	926 (30 %)	144 (42 %)	782 (28 %)	
4 (eGFR 15–29 mL/min/1.73 m^2^)	255 (8 %)	117 (34 %)	138 (5 %)	
Serum Hematocrit, mean (SD)	37.2 (4.6)	35.6 (4.9)	37.4 (4.6)	*P* <0.0001
Diabetes on problem list, N (%)	851 (27 %)	143 (42 %)	708 (26 %)	*P* <0.0001
Hypertension on problem list, N (%)	2099 (67 %)	254 (75 %)	1845 (66 %)	*P* = 0.003
Tobacco Use, N (%)				*P* = 0.02
Current	179 (8 %)	33 (12 %)	146 (7 %)	
Former	960 (41 %)	107 (38 %)	853 (41 %)	
Never	1224 (52 %)	143 (51 %)	1081 (52 %)	
Insurance status, N (%)				*P* = 0.002
Public	2391 (77 %)	252 (74 %)	2139 (77 %)	
Private	620 (20 %)	66 (19 %)	554 (20 %)	
Self Pay	107 (4 %)	23 (7 %)	84 (3 %)	
PCP visits yearly, mean (SD)	4.0 (2.9)	5.0 (3.6)	3.9 (2.8)	*P* <0.0001
Nephrology visits yearly, mean (SD)	N/A	2.3 (1.5)	N/A	N/A

### Nephrology co-management

Of the 3,118 stage 3 and 4 CKD patients, 341 (11 %) had at least one visit with a nephrologist during 2009 [191 (7.5 %) of stage 3 patients and 94 (50 %) of stage 4 patients]. On average, patients saw nephrology twice during the year (Table 1). Nephrology co-management was associated with younger age, male gender, Black or Hispanic race/ethnicity, hypertension, diabetes, and more frequent PCP visits (Table 1). Within the stage 4 CKD subgroup, the only covariates associated with nephrology co-management were younger age and more frequent PCP visits.

### Outcome measures

Patients co-managed with nephrology were more likely to have received tests monitoring for progression: serum eGFR and urine protein/albumin (Table 2). We found no evidence that PCP diagnosis of early CKD was responsible for these differences.

**Table 2:** Association of nephrology co-management with quality of care for pooled stage 3 and stage 4 CKD patients

	Unadjusted estimates^a^			Adjusted estimates^b^		
Outcome	Nephrology co-management (*n* = 341)	Solo PCP management (*n* = 2777)	*p* value	Nephrology co-management	Solo PCP management	*p* value
Serum eGFR^c^	100 %	93 %	*P* < 0.0001	98 %	94 %	*P* < 0.0001
Urine protein	87 %	37 %	*P* <0.0001	82 %	36 %	*P* < 0.0001
ACE/ARB prescription	81 %	65 %	*P* < 0.0001	77 %	69 %	*P* = 0.03
BP <140/90	68 %	72 %	*P* = 0.15	70 %	73 %	*P* = 0.41
BP <130/80	47 %	45 %	*P* = 0.40	47 %	46 %	*P* = 0.68
	Mean	Mean		Weighted estimate	Weighted estimate	
Systolic, mmHg	133.1	132.4	*P* = 0.54	132.6	130.8	*P* = 0.15
Diastolic, mmHg	70.9	72.1	*P* = 0.13	69.0	70.2	*P* = 0.12

^a^All estimates account for clustering by PCP

^b^Weighted percentage and p value estimated by multivariate model accounting for clustering by PCP and adjusting for age, gender, race/ethnicity, eGFR, hypertension, diabetes, and number of PCP visits

^c^Linear model due to 100 % rate in co-management group

Patients who were co-managed were more likely to receive an ACE/ARB prescription (Table 2). The mean BP was 133/72 mmHg. There were no significant associations between nephrology co-management and mean BP before or after adjustment (Table 2). With a BP goal of 140/90 mmHg, 71 % were under control and the likelihood of being under control was not associated with nephrology co-management. With a BP goal of 130/80 mmHg, 45 % were under control and the likelihood of BP being under control was not associated with nephrology co-management.

In stage 3 patients, nephrology co-management was associated with tests monitoring for progression (serum eGFR and urine protein/albumin), tests monitoring for complications (serum hemoglobin, serum calcium, and serum phosphorus), both before and after adjustment for potential confounders (Table 3). Co-management was associated with serum LDL testing before adjustment only. Patients who were co-managed were more likely to receive an ACE/ARB prescription. There were no differences in BP outcomes.

**Table 3:** Association of nephrology co-management with quality of care measures for CKD patients, stage 3 only

	Unadjusted estimates^a^			Adjusted estimates^b^		
Outcome	Nephrology co-management (*n* = 191)	Solo PCP management (*n* = 2363)	*p* value	Nephrology co-management	Solo PCP management	*p* value
Serum eGFR^c^	100 %	93 %	*P* < 0.0001	98 %	94 %	*P* < 0.0001
Urine protein	88 %	36 %	*P* < 0.0001	85 %	34 %	*P* < 0.0001
ACE/ARB prescription	84 %	65 %	*P* < 0.0001	79 %	69 %	*P* = 0.02
BP <140/90 mmHg	71 %	71 %	*P* = 0.93	71 %	72 %	*P* = 0.68
BP <130/80 mmHg	48 %	45 %	*P* = 0.36	46 %	45 %	*P* = 0.96
Serum LDL	83 %	77 %	*P* = 0.02	79 %	78 %	*P* = 0.74
Serum Hemoglobin or Hematocrit	96 %	79 %	*P* < 0.0001	96 %	83 %	*P* < 0.0001
Serum Calcium	99 %	91 %	*P* < 0.0001	99 %	93 %	*P* = 0.0002
Serum Phosphorus	81 %	18 %	*P* < 0.0001	76 %	17 %	*P* < 0.0001
	Mean	Mean		Weighted estimate	Weighted estimate	
Systolic, mmHg	132.8	132.5	*P* = 0.81	133.8	132.1	*P* = 0.21
Diastolic, mmHg	72.7	72.1	*P* = 0.57	71.9	72.1	*P* = 0.84

^a^All estimates account for clustering by PCP

^b^Percentage and p value estimated by multivariate model accounting for clustering by PCP and adjusting for age, gender, race/ethnicity, eGFR, hypertension, diabetes, and number of PCP visits

^c^Linear model due to 100 % rate in co-management group

In stage 4 patients, nephrology co-management was associated with a higher rate of one test monitoring for progression (urine protein/albumin), and tests monitoring for complications (serum hemoglobin, serum phosphorus, and serum parathyroid hormone), both before and after adjustment for potential confounders (Table 4). Co-management was associated with serum calcium testing before adjustment only (*p* = 0.04). Co-management was associated with mean diastolic blood pressure after adjustment only (*p* = 0.0007). Nephrology co-management was not associated with ACE/ARB prescription, mean systolic blood pressure, or blood pressure control.

**Table 4:** Association of nephrology co-management with quality of care measures for CKD patients, stage 4 only

	Unadjusted estimates^a^			Adjusted estimates^b^		
Outcome	Nephrology co-management (*n* = 94)	Solo PCP management (*n* = 95)	*p* value	Nephrology co-management	Solo PCP management	*p* value
Serum eGFR^c^	100 %	97 %	*P* = 0.08	100 %	97 %	*P* = 0.09
Urine protein	86 %	60 %	*P* < 0.0001	88 %	56 %	*P* < 0.0001
ACE/ARB prescription	77 %	72 %	*P* = 0.41	80 %	73 %	*P* = 0.26
BP <140/90 mmHg	64 %	69 %	*P* = 0.51	64 %	70 %	*P* = 0.52
BP <130/80 mmHg	46 %	47 %	*P* = 0.97	48 %	44 %	*p* = 0.59
Serum LDL	76 %	73 %	*P* = 0.59	77 %	80 %	*P* = 0.69
Serum Hemoglobin or Hematocrit^c^	99 %	91 %	*P* = 0.01	99 %	91 %	*P* = 0.04
Serum Calcium^c^	100 %	96 %	*P* = 0.04	100 %	96 %	*P* = 0.05
Serum Phosphorus	90 %	49 %	*P* < 0.0001	91 %	50 %	*P* < 0.0001
Serum PTH	92 %	32 %	*P* < 0.0001	92 %	33 %	*P* < 0.0001
	Mean	Mean		Weighted estimate	Weighted estimate	
Systolic, mmHg	132.3	131.7	*P* = 0.85	130.6	130.0	*p* = 0.84
Diastolic, mmHg	67.7	71.2	*P* = 0.06	64.6	69.9	*P* = 0.0007

^a^All estimates account for clustering by PCP

^b^Percentage and p value estimated by multivariate model accounting for clustering by PCP and adjusting for age, gender, race/ethnicity, eGFR, hypertension, diabetes, and number of PCP visits. Race/ethnicity categories were collapsed to White, Black, Other due to inability to perform logistic regression with small cells

^c^Linear model due to 100 % rate in co-management group

## Discussion

We found that only a small proportion (8 %) of stage 3 CKD patients and half of stage 4 CKD patients were co-managed by nephrology. Co-management was associated with socio-demographic differences, particularly in stage 3 CKD patients for whom co-management was associated with younger age, male gender and minority race/ethnicity. Co-management was associated with diabetes, hypertension, and more frequent PCP visits. After controlling for these potential confounders, co-management was associated with monitoring tests, both for progression and for complications. Co-management was associated with higher rates of ACE/ARB prescription in stage 3 CKD, but not in stage 4 CKD. Co-management was not associated with higher rates of cardiovascular risk modification through lipid monitoring or blood pressure control.

Our finding of a difference between the two groups for ACE/ARB prescription in stage 3, though not in stage 4, is in concert with another recently published study from the Chronic Renal Insuffiency Cohort (CRIC) [15]. One explanation for the higher impact of nephrology co-management in stage 3 CKD as compared to stage 4 CKD is low PCP recognition of CKD in stage 3. As we showed in a prior study, PCPs are more likely to diagnose CKD in patients with more advanced disease [16].

Co-management was associated with age, gender, and race/ethnicity. These associations align with patients who have higher muscle mass. This may indicate PCPs are still using serum creatinine levels rather than eGFR to judge severity of CKD in early disease. The only socio-demographic characteristic associated with nephrology referral in stage 4 CKD was younger age. PCPs were more likely to refer patients with diabetes in the stage 3 subgroup, which may reflect a higher rate of urine albumin screening and appropriate subsequent referral of albuminuric patients [16]. Patients who saw their PCP less often were less likely to be referred, which may reflect competing demands during office visits [17, 18]. We saw a similar dose–response relationship between the number of primary care visits and CKD documentation in our prior study [16] and the AVENIR study found a dose–response relationship between the number of nephrology visits and quality of care [19].

Other primary care CKD studies have revealed suboptimal urine protein testing, serum phosphorus testing, serum parathyroid hormone testing, prescription of ACE/ARB, and blood pressure control [16, 20–22]. The AVENIR nephrology clinic study reported low rates, similar to ours, of urine protein testing at 50 % and ACE/ARB prescription at 67 %, in addition to a  BP control rate of 14 % (<130/80 mmHg), lower than ours [19]. One U.S. study designed to examine the impact of automated eGFR reporting also compared co-managed and solo managed patients (in stage 3b and 4, or eGFR 15–45 mL/min/1.73 m^2^). They found a significant difference in serum hemoglobin testing, serum phosphorus testing, and serum PTH testing, but neither urine protein testing (with a rate of <30 % overall) nor ACE/ARB prescribing (with a rate of <60 % overall) [23]. Ours is the first study to examine blood pressure outcomes in a similar manner.

There are several limitations to this study. There is a potential for type II error when we conclude that there is no difference in BP control between groups because co-managed patients may be sicker than solo managed patients. An analysis of anti-hypertensive regimens would help to elucidate any difference in management. There may be other patient level confounders for which we were unable to adjust. ACE/ARB prescriptions were assessed in all CKD patients, though these are only recommended for patients with either hypertension, diabetic kidney disease, or proteinuria. Our laboratory uses the MDRD equation to calculate eGFR without accounting for race. Generalizability is limited by the fact that the population is limited to one primary care network. We were unable to measure clinical outcomes such as progression to ESRD and mortality.

Our findings have implications for the following: 1) PCP solo management, 2)  nephrology co-management, and developing a systematic approach to  referral to nephrology.

First, PCPs should improve solo management in stage 3 CKD through monitoring for complications, monitoring for progression of disease, and intervening to delay progression. One successful approach is through point-of-care EHR alerts that remind the physician of the diagnosis and recommended management of stage 3 CKD. Such reminders have improved urine protein testing [24]. A second approach which addresses the lack of time in primary care visits is population management by a non-physician. Nurse-led population management has improved quality of care for diabetes and hypertension [25, 26]. A study in one of our practices combined EHR alerts and population management to successfully improve PCP management of CKD [27].

Second, quality of care for co-managed patients could improve as well. Co-management did not increase the likelihood of cardiovascular risk modification in stage 3 or stage 4 CKD. PCPs and nephrologists working together should be able to control patients’ blood pressure, yet we saw no difference in this measure between solo managed and co-managed patients. Both EHR alerts and population management are likely to be part of the answer [26, 28], but to date, there have been no successful systems-level interventions for uncontrolled blood pressure in a CKD population. Ideally, we will develop EHR alerts that are sophisticated enough to track management over time and to consider previous medication regimens and drug allergies [29, 30]. Perhaps more importantly, we should routinely employ effective patient-centered interventions to improve medication adherence [31, 32].

Third, and just as critical as the first (i.e. improving PCP solo management) and the second (i.e. improving co-management), is developing a systematic approach to referral. The current approach relies on  creatinine-based estimates of kidney function that do not accurately predict whether and when patients might progress [38, 39]; many patients do not progress.

That being said, two studies showed a slowing of progression in stage 3 CKD when nephrologists monitored patients electronically and gave advice to PCPs [40, 41]. At this point, we only have two studies to answer the important question of whether we can delay or prevent progression to ESRD through comanagement in stage 3 CKD. Further studies would be welcome. A potentially better approach would take into account the fact that nephrology referral has a mortality benefit when done 12 to 72 months before initiating dialysis [33–37]. PCPs may be aided by risk estimation models such as one validated by Tangri and colleagues that incorporates additional metabolic parameters beyond serum creatinine in order to predict how likely a patient is to progress to renal failure within a five years [42]. Such prediction models could encourage PCPs to increase appropriate referrals to nephrology based on length of time before renal replacement therapy is needed, rather than stage-based referral.

## Conclusions

In conclusion, early CKD is commonly managed in primary care, but patients who are co-managed by nephrology receive better monitoring for progression and complications. Improving the quality of PCP solo management will help to close this gap. In addition, about one-quarter of early CKD patients have uncontrolled blood pressure whether they have been referred to a nephrologist or not. We should study interventions to improve co-management of blood pressure and to decrease cardiovascular events. These interventions should include population management, EHR tools, and patient-centered interventions. Finally, we have sophisticated tools to risk-stratify patients with CKD and we must learn how best to employ these tools in primary care practices to systematize referral of high-risk patients.

## Abbreviations

CKD: Chronic kidney disease
PCP: Primary care physician
EHR: Electronic health record
eGFR: Estimated glomerular filtration rate
ACE: Angiotensin converting enzyme inhibitor
ARB: Angiotensin receptor blocker
BP: Blood pressure

## References

[1] Mandell BF (2014). The generalist, the specialist, and the patient with chronic kidney disease. Cleve Clin J Med.

[2] Kidney Disease: Improving Global Outcomes (KDIGO) CKD Work Group (2013). KDIGO 2012 clinical practice guideline for the evaluation and management of chronic kidney disease. Kidney Int Suppl.

[3] Black C, Sharma P, Scotland G, McCullough K, McGurn D, Robertson L (2010). Early referral strategies for management of people with markers of renal disease: a systematic review of the evidence of clinical effectiveness, cost-effectiveness and economic analysis. Health Technol Assess.

[4] Smart NA, Titus TT (2011). Outcomes of early versus late nephrology referral in chronic kidney disease: a systematic review. Am J Med.

[5] National Kidney Foundation (2002). K/DOQI clinical practice guidelines for chronic kidney disease: evaluation, classification, and stratification. Am J Kidney Dis.

[6] Wright A, Pang J, Feblowitz JC, Maloney FL, Wilcox AR, McLoughlin KS (2012). Improving completeness of electronic problem lists through clinical decision support: a randomized, controlled trial. J Am Med Inform Assoc.

[7] Kidney Disease Outcomes Quality Initiative (K/DOQI) (2004). K/DOQI clinical practice guidelines on hypertension and antihypertensive agents in chronic kidney disease. Am J Kidney Dis.

[8] James PA, Oparil S, Carter BL, Cushman WC, Dennison-Himmelfarb C, Handler J (2014). 2014 evidence-based guideline for the management of high blood pressure in adults: report from the panel members appointed to the eighth joint national committee (JNC 8). JAMA.

[9] Kidney Disease: Improving Global Outcomes (KDIGO) Blood Pressure Work Group (2012). KDIGO clinical practice guideline for the management of blood pressure in chronic kidney disease. Kidney Int Suppl.

[10] Taler SJ, Agarwal R, Bakris GL, Flynn JT, Nilsson PM, Rahman M (2013). KDOQI US commentary on the 2012 KDIGO clinical practice guideline for management of blood pressure in CKD. Am J Kidney Dis.

[11] Uhlig K, Berns JS, Kestenbaum B, Kumar R, Leonard MB, Martin KJ (2010). KDOQI US commentary on the 2009 KDIGO clinical practice guideline for the diagnosis, evaluation, and treatment of CKD-mineral and bone disorder (CKD-MBD). Am J Kidney Dis.

[12] Kidney Disease: Improving Global Outcomes (KDIGO) Anemia Work Group (2012). KDIGO clinical practice guideline for anemia in chronic kidney disease. Kidney Int Suppl (2011).

[13] Kidney Disease: Improving Global Outcomes (KDIGO) CKD-MBD Work Group (2009). KDIGO clinical practice guideline for the diagnosis, evaluation, prevention, and treatment of chronic kidney disease-mineral and bone disorder (CKD-MBD). Kidney Int Suppl.

[14] Kliger AS, Foley RN, Goldfarb DS, Goldstein SL, Johansen K, Singh A (2013). KDOQI US commentary on the 2012 KDIGO clinical practice guideline for anemia in CKD. Am J Kidney Dis.

[15] Ricardo AC, Roy JA, Tao K, Alper A, Chen J, Drawz PE, et al. Influence of nephrologist care on management and outcomes in adults with chronic kidney disease. J Gen Intern Med. 2015 Jul 3. [Epub ahead of print]. 10.1007/s11606-015-3452-x PMC4700009 26138006

[16] Samal L, Linder JA, Bates DW, Wright A (2014). Electronic problem list documentation of chronic kidney disease and quality of care. BMC Nephrol.

[17] Parchman ML, Pugh JA, Romero RL, Bowers KW (2007). Competing demands or clinical inertia: the case of elevated glycosylated hemoglobin. Ann Fam Med.

[18] Ostbye T, Yarnall KS, Krause KM, Pollak KI, Gradison M, Michener JL (2005). Is there time for management of patients with chronic diseases in primary care?. Ann Fam Med.

[19] Thilly N, Boini S, Kessler M, Briancon S, Frimat L (2009). Chronic kidney disease: appropriateness of therapeutic management and associated factors in the AVENIR study. J Eval Clin Pract.

[20] Philipneri MD, Rocca Rey LA, Schnitzler MA, Abbott KC, Brennan DC, Takemoto SK (2008). Delivery patterns of recommended chronic kidney disease care in clinical practice: administrative claims-based analysis and systematic literature review. Clin Exp Nephrol.

[21] Litvin CB, Nietert PJ, Wessell AM, Jenkins RG, Ornstein SM (2011). Recognition and management of CKD in primary care. Am J Kidney Dis.

[22] Allen AS, Forman JP, Orav EJ, Bates DW, Denker BM, Sequist TD (2011). Primary care management of chronic kidney disease. J Gen Intern Med.

[23] Abdel-Kader K, Fischer GS, Johnston JR, Gu C, Moore CG, Unruh ML (2011). Characterizing pre-dialysis care in the era of eGFR reporting: a cohort study. BMC Nephrol.

[24] Abdel-Kader K, Fischer GS, Li J, Moore CG, Hess R, Unruh ML (2011). Automated clinical reminders for primary care providers in the care of CKD: a small cluster-randomized controlled trial. Am J Kidney Dis.

[25] Maclean CD, Gagnon M, Callas P, Littenberg B (2009). The Vermont diabetes information system: a cluster randomized trial of a population based decision support system. J Gen Intern Med.

[26] Jaffe MG, Lee GA, Young JD, Sidney S, Go AS (2013). Improved blood pressure control associated with a large-scale hypertension program. JAMA.

[27] Mendu ML, Schneider LI, Aizer AA, Singh K, Leaf DE, Lee TH (2014). Implementation of a CKD checklist for primary care providers. Clin J Am Soc Nephrol.

[28] Samal L, Linder JA, Lipsitz SR, Hicks LS (2011). Electronic health records, clinical decision support, and blood pressure control. Am J Manag Care.

[29] Feblowitz JC, Wright A, Singh H, Samal L, Sittig DF (2011). Summarization of clinical information: a conceptual model. J Biomed Inform.

[30] Samal L, Wright A, Wong BT, Linder JA, Bates DW (2011). Leveraging electronic health records to support chronic disease management: the need for temporal data views. Inform Prim Care.

[31] Grant RW, Pandiscio JC, Pajolek H, Woulfe A, Pelletier A, Kvedar J (2012). Implementation of a web-based tool for patient medication self-management: the medication self-titration evaluation programme (med-STEP) for blood pressure control. Inform Prim Care.

[32] Haynes RB, Ackloo E, Sahota N, McDonald HP, Yao X (2008). Interventions for enhancing medication adherence. Cochrane Database Syst Rev.

[33] Kinchen KS, Sadler J, Fink N, Brookmeyer R, Klag MJ, Levey AS (2002). The timing of specialist evaluation in chronic kidney disease and mortality. Ann Intern Med.

[34] de Jager DJ, Voormolen N, Krediet RT, Dekker FW, Boeschoten EW, Grootendorst DC (2011). Association between time of referral and survival in the first year of dialysis in diabetics and the elderly. Nephrol Dial Transplant.

[35] Kim do H, Kim M, Kim H, Kim YL, Kang SW, Yang CW (2013). Early referral to a nephrologist improved patient survival: prospective cohort study for end-stage renal disease in Korea. PLoS One.

[36] Khan SS, Xue JL, Kazmi WH, Gilbertson DT, Obrador GT, Pereira BJ (2005). Does predialysis nephrology care influence patient survival after initiation of dialysis?. Kidney Int.

[37] Jungers P, Massy ZA, Nguyen-Khoa T, Choukroun G, Robino C, Fakhouri F (2001). Longer duration of predialysis nephrological care is associated with improved long-term survival of dialysis patients. Nephrol Dial Transplant.

[38] Li L, Astor BC, Lewis J, Hu B, Appel LJ, Lipkowitz MS (2012). Longitudinal progression trajectory of GFR among patients with CKD. Am J Kidney Dis.

[39] O’Hare AM, Batten A, Burrows NR, Pavkov ME, Taylor L, Gupta I (2012). Trajectories of kidney function decline in the 2 years before initiation of long-term dialysis. Am J Kidney Dis.

[40] Lee BJ, Forbes K (2009). The role of specialists in managing the health of populations with chronic illness: the example of chronic kidney disease. BMJ.

[41] Lee B, Turley M, Meng D, Zhou Y, Garrido T, Lau A (2012). Effects of proactive population-based nephrologist oversight on progression of chronic kidney disease: a retrospective control analysis. BMC Health Serv Res.

[42] Tangri N, Stevens LA, Griffith J, Tighiouart H, Djurdjev O, Naimark D (2011). A predictive model for progression of chronic kidney disease to kidney failure. JAMA.

